# (Butane-1,4-di­yl)(trimethyl­phosphane-κ*P*)[tris­(3,5-dimethyl­pyrazol-1-yl-κ*N* ^2^)hydro­borato]iridium(III)

**DOI:** 10.1107/S1600536813008040

**Published:** 2013-04-05

**Authors:** Margarita Gómez, Laura L. Santos, Margarita Paneque, Kurt Mereiter

**Affiliations:** a Instituto de Investigaciones Químicas (IIQ) and Departamento de Química Inorgánica, Consejo Superior de Investigaciones Cientificas (CSIC) and Universidad de Sevilla, Avenida Américo Vespucio 49, 41092 Sevilla, Spain; b Institute of Chemical Technologies and Analytics, Vienna University of Technology, Getreidemarkt 9/164SC, A-1060 Vienna, Austria

## Abstract

In the mononuclear title iridium(III) complex, [Ir(C_4_H_8_)(C_15_H_22_BN_6_)(C_3_H_9_P)], which is based on the [tris­(3,5-dimethyl­pyrazol-1-yl)hydro­borato]iridium moiety, Ir[Tp^Me2^], the Ir^III^ atom is coordinated by a chelating butane-1,4-diyl fragment and a trimethyl­phosphane ligand in a modestly distorted octa­hedral coordination environment formed by three facial N, two C and one P atom. The iridium–butane-1,4-diyl ring has an envelope conformation. This ring is disordered because alternately the second or the third C atom of the butane-1,4-diyl fragment function as an envelope flap atom (the occupancy ratio is 1:1). In the crystal, mol­ecules are organized into densely packed columns extending along [101]. Coherence between the mol­ecules is essentially based on van der Waals inter­actions.

## Related literature

For general aspects of hydrogen tris­pyrazolylborate ligands, see: Pettinari & Trofimenko (2008 ▶). For general information on mechanistic aspects of organometallic reactions, involving oxidative addition and reductive elimination, see: Crabtree (2005 ▶). For information on σ-CAM mechanisms, see: Perutz & Sabo-Etienne (2007 ▶). For general information on the chemistry and potential of Ir[Tp^Me2^] complexes, see: Conejero *et al.* (2010 ▶). For selected aspects of the synthesis and the crystal structure of the precursor of the title compound, see: Paneque *et al.* (2000 ▶). For aspects of the chemistry of a CO- instead of PMe_3_-containing analogue to the precursor of the title compound, see: Gómez *et al.* (2007 ▶). For a description of the Cambridge Structural Database, see: Allen (2002 ▶).
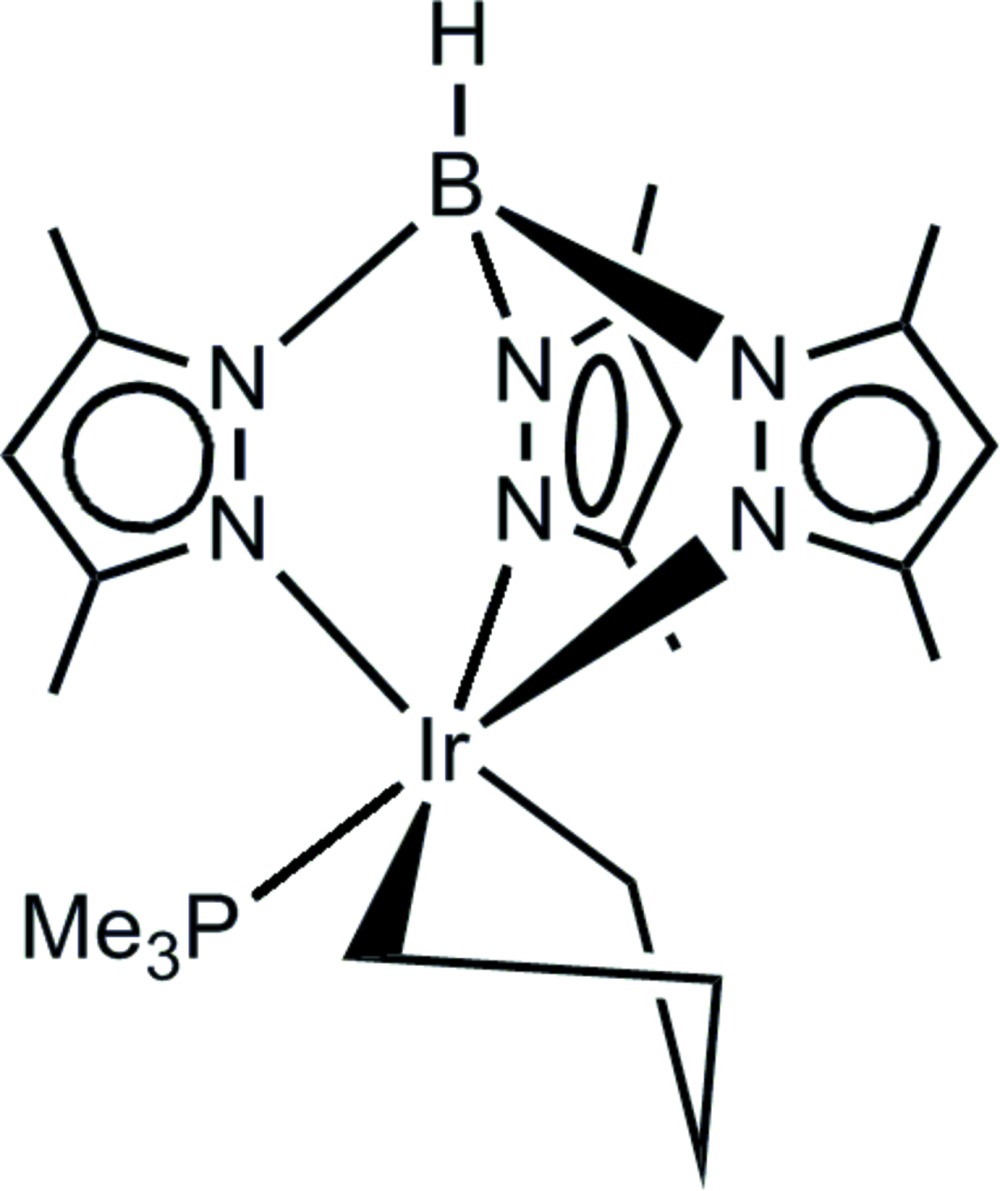


## Experimental

### 

#### Crystal data

[Ir(C_4_H_8_)(C_15_H_22_BN_6_)(C_3_H_9_P)]
*M* *_r_* = 621.57
Monoclinic, 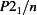

*a* = 11.1865 (5) Å
*b* = 18.1771 (8) Å
*c* = 13.4748 (6) Å
β = 112.883 (1)°
*V* = 2524.3 (2) Å^3^
*Z* = 4
Mo *K*α radiation
μ = 5.37 mm^−1^
*T* = 173 K
0.22 × 0.15 × 0.14 mm

#### Data collection

Bruker SMART APEX CCD diffractometer
Absorption correction: multi-scan (*SADABS*; Bruker, 2003 ▶) *T*_min_ = 0.36, *T*_max_ = 0.47
37102 measured reflections
7359 independent reflections
6892 reflections with *I* > 2σ(*I*)
*R*_int_ = 0.018

#### Refinement

*R*[*F* ^2^ > 2σ(*F* ^2^)] = 0.017
*wR*(*F* ^2^) = 0.040
*S* = 1.08
7359 reflections
298 parameters
25 restraints
H-atom parameters constrained
Δρ_max_ = 0.86 e Å^−3^
Δρ_min_ = −0.32 e Å^−3^

### 

Data collection: *SMART* (Bruker, 2003 ▶); cell refinement: *SAINT* (Bruker, 2003 ▶); data reduction: *SAINT*; program(s) used to solve structure: *SHELXS97* (Sheldrick, 2008 ▶); program(s) used to refine structure: *SHELXL97* (Sheldrick, 2008 ▶); molecular graphics: *Mercury* (Macrae *et al.*, 2006 ▶); software used to prepare material for publication: *publCIF* (Westrip, 2010 ▶).

## Supplementary Material

Crystal structure: contains datablock(s) I, global. DOI: 10.1107/S1600536813008040/gk2565sup1.cif

Structure factors: contains datablock(s) I. DOI: 10.1107/S1600536813008040/gk2565Isup2.hkl

Additional supplementary materials:  crystallographic information; 3D view; checkCIF report

## Figures and Tables

**Table 1:** Selected bond lengths (Å)

Ir1—C19*A*	2.0780 (18)
Ir1—C16*A*	2.0871 (19)
Ir1—N5	2.1781 (14)
Ir1—N3	2.2233 (15)
Ir1—P1	2.2381 (5)
Ir1—N1	2.2590 (15)

**Fig. 1.**
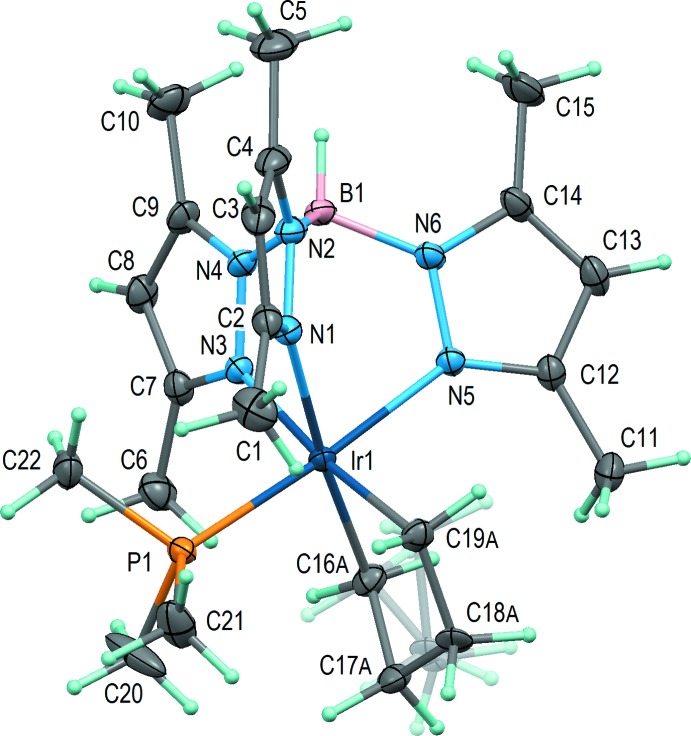
The asymmetric unit of the title compound with displacement ellipsoids drawn at the 30% probability level showing the disordered butane-1,4-diyl fragment C16—C17—C18—C19 in both conformations (A-conformation dark, B-conformation pale), see text.

**Fig. 2.**
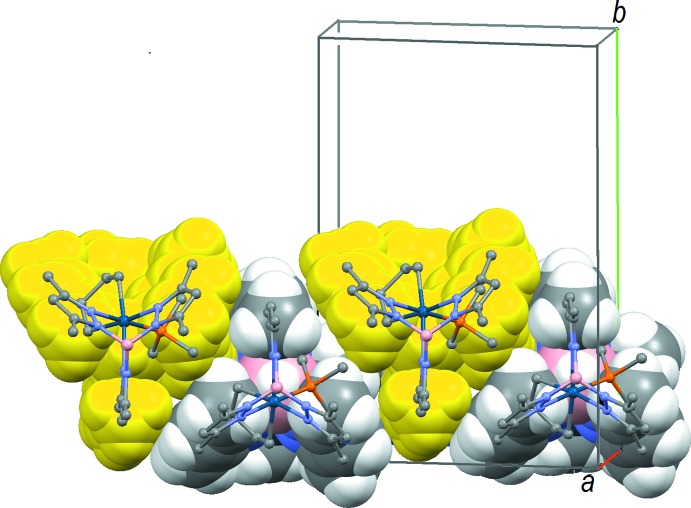
Stacking of the molecules of I to form efficiently packed columns parallel to [101].

## supplementary crystallographic information

### Comment

Iridium complexes with the stabilizing ligand hydrogen tris(3,5-dimethylpyrazolyl)borate (Tp^Me2^) (Pettinari & Trofimenko, 2008) and containing carbon bearing co-ligands possess a rich chemistry (Conejero *et al.*, 2010) and are capable of a broad range of bond activation and coupling reactions either *via* oxidative addition and reductive elimination (Crabtree, 2005) or *via*σ-CAM mechanisms (Perutz & Sabo-Etienne, 2007). When the conveniently accessible Ir(I) complex [(Tp^Me2^)Ir(η^4^-CH_2_═CH—CH═CH_2_)] containing a π-bonded butadiene is treated with a Lewis base like PMe_3_ it transforms cleanly into the Ir(III) complex [(Tp^Me2^)Ir(κ^2^-CH_2_—CH═CH—CH_2_)(PMe_3_)] in a process which implies the transformation of the butadiene ligand into a but-2-ene-1,4-diyl one (Paneque *et al.*, 2000). This complex is inert to substitution of the phosphine ligand so the metallacyclic structure can experience some cyclopentene-like reactivity without rupturing the Ir—C bonds. The analogous product with a CO ligand instead of PMe_3_ behaves similarly and experiences for instance a series of reactions (subsequent hydroboration-oxidation of double bond, alcohol oxidation to ketone and α-formylation of ketone) leading to the formation a new α-formyl-3-iridacyclopentanone (Gómez *et al.*, 2007). The catalytic hydrogenation of the PMe_3_ derivative under slightly harsher conditions leads to the formation of the title compound [(Tp^Me2^)Ir(κ^2^-CH_2_—CH_2_—CH_2_—CH_2_)(PMe_3_)], (I). A view of the complex is shown in Fig. 1. Iridium has a modestly distorted octahedral coordination with bond lengths given in Table 1. Bond angles about Ir are in the ranges 82.12 (8)–96.06 (7)° and 172.64 (7)–175.42 (4)° with the smallest angle (C—Ir—C = 82.12 (8)°) for the Ir butane-1,4-diyl ring. This ring has an envelope conformation but is disordered because either the 2^nd^ or the 3^rd^ carbon atom of the butane fragment is the flap atom in 1:1 ratio (*cf.* Fig. 1). In the first case Ir1, C16A, C18A, and C19A are flat within 0.053 Å mean deviation from planarity and C17A is the flap atom that deviates by 0.569 (6) Å from the mean plane of the four. In the second case Ir1, C16B, C17B, and C19B are flat (0.028 Å mean deviation) and C18B is the flap atom which deviates by 0.537 (7) Å from the corresponding mean plane. Except for the envelope conformation of the IrC_4_ ring the molecular structure of I is closely similar to that of [(Tp^Me2^)Ir(κ^2^-CH_2_—CH═CH—CH_2_)(PMe_3_)] (Paneque *et al.*, 2000; for crystal structure data see Cambridge Crystallographic Database, refcode ABIBOC; CCD version 5.33; Allen, 2002). This includes also bond lengths and bond angles about Ir, which for the latter complex are: Ir—N = 2.25 (1), 2.22 (1), 2.16 (1) Å; Ir—C = 2.09 (1), 2.09 (1) Å, Ir—P = 2.234 (3) Å; the C═C bond in the olefinic IrC_4_ ring is 1.27 (2) Å compared with C—C = 1.536 (1) Å for the aliphatic IrC_4_ ring in I. Despite similar shapes of the two complexes under consideration and analogous crystallization conditions (see Experimental) their crystal structures are dissimilar: [(Tp^Me2^)Ir(κ^2^-CH_2_—CH═CH—CH_2_)(PMe_3_)] crystallized as a dichloromethane solvate of triclinic symmetry (Paneque *et al.*, 2000), whereas I is monoclinic and unsolvated. In the lack of distinctly polar groups on the outer surface of the molecules of I, their crystal structure is held together essentially by van der Waals interactions. The most prominent feature of the crystal structure of I are columns of tightly packed molecules extending along [101], as exemplified in Fig. 2. Mutual contacts between the columns perpendicular to [101] are more open and appear to represent weaker coherence. In the crystal structure of [(Tp^Me2^)Ir(κ^2^-CH_2_—CH═CH—CH_2_)(PMe_3_)].1/4CH_2_Cl_2_ (Paneque *et al.*, 2000) the column motif of I is absent.

### Experimental

A solution of [(Tp^Me2^)Ir(κ^2^-CH_2_—CH═CH—CH_2_)(PMe_3_)] (0.10 g, 0.16 mmol; for synthesis see Paneque *et al.*, 2000) in dioxane (10 ml), with a small amount of PtO_2_ as catalyst, was transferred to a pressure vessel, charged with H_2_ at 4 bar and heated at 353 K for 4 days. Then the solvent was removed under reduced pressure and the crude product was purified by column chromatography (silicagel, hexane/Et_2_O 10/1 *v*/*v*) to give the title compound I in 44% yield. Crystallization from hexane/CH_2_Cl_2_ (1:2) at 253 K gave I as colourless crystals. ^1^H NMR (CDCl_3_, 298 K) δ 5.71, 5.68 (s, 2:1, 3 CH_pz_), 2.44, 2.37, 2.36, 2.26 (s, 1:2:2:1, 6 Me_pz_), 2.33 (m, 4 H, 2 CH_A_H_B_), 1.64, 1.27 (m, 2 H cada, 2 CH_C_H_D_), 1.37 (d, 9 H, ^2^*J*_HP_ = 9.2 Hz, PMe_3_). ^13^C{^1^H} NMR (CDCl_3_, 298 K) δ 150.5, 148.9 (d, *J*_CP_ = 3 Hz) (2:1:2:1, C_qpz_), 108.3 (d, *J*_CP_ = 4 Hz), 107.9 (1:2 CH_pz_), 34.8 (C^2^, ^3^*J*_CP_ = 2 Hz, ^1^*J*_CH_ = 122 Hz), 16.2 (d, ^1^*J*_CP_ = 37 Hz, PMe_3_), 16.0, 15.1, 13.7, 13.4 (2:1:1:2, Me_pz_), -3.6 (C1, ^2^*J*_CP_ = 8 Hz, ^1^*J*_CH_ = 125 Hz). ^31^P {^1^H} NMR (CDCl_3_, 298 K) δ -48.0 p.p.m..

### Refinement

Conformational disorder in the 5-membered Ir-butane-1,4-diyl chelate ring was resolved with split positions (1:1; ratio fixed after preceeding refinement had indicated this) for the 2,3-carbon atoms (C17A/C17B and C18A/C18B) stabilized by a SADI restraint for all C—C bonds, EXYZ constraints (C16A/C16B, C19A/C19B), EADP constraints (C16A/C16B, C18A/C18B, C19A/C19B), and a DELU 0.001 0.001 restraint for C16A through C19B. H atoms were placed in calculated positions and thereafter treated as riding, C—H = 0.95–0.99 Å, B—H = 1.00 Å, *U*i_so_(H) = 1.2–1.5*U*_eq_(C,B), using AFIX 137 of program *SHELXL97* (Sheldrick, 2008) for the methyl groups.

### Crystal data

**Table:**

[Ir(C_4_H_8_)(C_15_H_22_BN_6_)(C_3_H_9_P)]	*F*(000) = 1240
*M**_r_* = 621.57	*D*_x_ = 1.636 Mg m^−^^3^
Monoclinic, *P*2_1_/*n*	Mo *K*α radiation, λ = 0.71073 Å
Hall symbol: -P 2yn	Cell parameters from 8250 reflections
*a* = 11.1865 (5) Å	θ = 2.3–30.0°
*b* = 18.1771 (8) Å	µ = 5.37 mm^−^^1^
*c* = 13.4748 (6) Å	*T* = 173 K
β = 112.883 (1)°	Oval, colourless
*V* = 2524.3 (2) Å^3^	0.22 × 0.15 × 0.14 mm
*Z* = 4	

### Data collection

**Table:**

Bruker SMART APEX CCD diffractometer	7359 independent reflections
Radiation source: fine-focus sealed tube	6892 reflections with *I* > 2σ(*I*)
Graphite monochromator	*R*_int_ = 0.018
Detector resolution: 0.11 pixels mm^-1^	θ_max_ = 30.0°, θ_min_ = 2.0°
ω and φ scans	*h* = −15→15
Absorption correction: multi-scan (*SADABS*; Bruker, 2003)	*k* = −25→25
*T*_min_ = 0.36, *T*_max_ = 0.47	*l* = −18→18
37102 measured reflections	

### Refinement

**Table:**

Refinement on *F*^2^	Primary atom site location: structure-invariant direct methods
Least-squares matrix: full	Secondary atom site location: difference Fourier map
*R*[*F*^2^ > 2σ(*F*^2^)] = 0.017	Hydrogen site location: inferred from neighbouring sites
*wR*(*F*^2^) = 0.040	H-atom parameters constrained
*S* = 1.08	*w* = 1/[σ^2^(*F*_o_^2^) + (0.0188*P*)^2^ + 1.5354*P*] where *P* = (*F*_o_^2^ + 2*F*_c_^2^)/3
7359 reflections	(Δ/σ)_max_ = 0.002
298 parameters	Δρ_max_ = 0.86 e Å^−^^3^
25 restraints	Δρ_min_ = −0.32 e Å^−^^3^

### Special details

**Table:**

Geometry. All e.s.d.'s (except the e.s.d. in the dihedral angle between two l.s. planes) are estimated using the full covariance matrix. The cell e.s.d.'s are taken into account individually in the estimation of e.s.d.'s in distances, angles and torsion angles; correlations between e.s.d.'s in cell parameters are only used when they are defined by crystal symmetry. An approximate (isotropic) treatment of cell e.s.d.'s is used for estimating e.s.d.'s involving l.s. planes.
Refinement. Refinement of *F*^2^ against ALL reflections. The weighted *R*-factor *wR* and goodness of fit *S* are based on *F*^2^, conventional *R*-factors *R* are based on *F*, with *F* set to zero for negative *F*^2^. The threshold expression of *F*^2^ > σ(*F*^2^) is used only for calculating *R*-factors(gt) *etc*. and is not relevant to the choice of reflections for refinement. *R*-factors based on *F*^2^ are statistically about twice as large as those based on *F*, and *R*- factors based on ALL data will be even larger.

### Fractional atomic coordinates and isotropic or equivalent isotropic displacement parameters (Å^2^)

**Table:**

	*x*	*y*	*z*	*U*_iso_*/*U*_eq_	Occ. (<1)
Ir1	0.330539 (6)	0.149427 (3)	0.140251 (5)	0.02163 (2)	
P1	0.13156 (4)	0.17885 (3)	0.02261 (4)	0.02555 (9)	
N1	0.41101 (15)	0.26249 (9)	0.13371 (12)	0.0279 (3)	
N2	0.52542 (16)	0.26511 (9)	0.11853 (13)	0.0313 (3)	
N3	0.39782 (14)	0.11256 (9)	0.01330 (12)	0.0271 (3)	
N4	0.50615 (16)	0.14845 (9)	0.01311 (13)	0.0289 (3)	
N5	0.52880 (14)	0.12008 (9)	0.24383 (12)	0.0258 (3)	
N6	0.62443 (15)	0.14889 (9)	0.21463 (13)	0.0299 (3)	
B1	0.5921 (2)	0.19391 (13)	0.11026 (17)	0.0312 (4)	
H1	0.6748	0.2061	0.1017	0.037*	
C1	0.2641 (3)	0.35688 (13)	0.1631 (3)	0.0507 (6)	
H1A	0.2366	0.3178	0.1997	0.076*	
H1B	0.1940	0.3674	0.0934	0.076*	
H1C	0.2842	0.4014	0.2076	0.076*	
C2	0.3816 (2)	0.33261 (11)	0.14579 (16)	0.0342 (4)	
C3	0.4767 (2)	0.37982 (12)	0.13857 (17)	0.0402 (5)	
H3	0.4791	0.4319	0.1446	0.048*	
C4	0.5652 (2)	0.33560 (13)	0.12097 (17)	0.0406 (5)	
C5	0.6899 (3)	0.35592 (16)	0.1088 (2)	0.0595 (8)	
H5A	0.6973	0.4096	0.1075	0.089*	
H5B	0.6894	0.3353	0.0415	0.089*	
H5C	0.7639	0.3360	0.1697	0.089*	
C6	0.2510 (2)	0.01374 (14)	−0.09982 (18)	0.0441 (5)	
H6A	0.2293	0.0053	−0.0370	0.066*	
H6B	0.2763	−0.0329	−0.1225	0.066*	
H6C	0.1753	0.0339	−0.1589	0.066*	
C7	0.36048 (18)	0.06676 (11)	−0.07123 (15)	0.0305 (4)	
C8	0.4424 (2)	0.07415 (12)	−0.12664 (16)	0.0360 (4)	
H8	0.4361	0.0486	−0.1900	0.043*	
C9	0.5333 (2)	0.12547 (13)	−0.07166 (16)	0.0345 (4)	
C10	0.6438 (3)	0.15446 (15)	−0.0960 (2)	0.0503 (6)	
H10A	0.6357	0.2080	−0.1049	0.075*	
H10B	0.6423	0.1320	−0.1626	0.075*	
H10C	0.7258	0.1425	−0.0365	0.075*	
C11	0.5281 (2)	0.04034 (13)	0.39711 (17)	0.0415 (5)	
H11A	0.4682	0.0728	0.4134	0.062*	
H11B	0.5949	0.0225	0.4645	0.062*	
H11C	0.4801	−0.0016	0.3544	0.062*	
C12	0.59054 (18)	0.08183 (11)	0.33483 (14)	0.0304 (4)	
C13	0.72443 (19)	0.08640 (13)	0.36404 (16)	0.0375 (4)	
H13	0.7898	0.0641	0.4247	0.045*	
C14	0.74271 (19)	0.12949 (14)	0.28780 (16)	0.0370 (4)	
C15	0.8676 (2)	0.15468 (18)	0.2836 (2)	0.0576 (8)	
H15A	0.8712	0.2085	0.2857	0.086*	
H15B	0.8734	0.1373	0.2168	0.086*	
H15C	0.9403	0.1348	0.3455	0.086*	
C16A	0.26125 (19)	0.04707 (11)	0.16388 (15)	0.0317 (4)	0.50
H16A	0.2210	0.0214	0.0938	0.038*	0.50
H16B	0.3346	0.0167	0.2114	0.038*	0.50
C17A	0.1607 (4)	0.0554 (2)	0.2149 (3)	0.0360 (8)	0.50
H17A	0.0758	0.0709	0.1603	0.043*	0.50
H17B	0.1492	0.0083	0.2471	0.043*	0.50
C18A	0.2157 (4)	0.11462 (19)	0.3022 (4)	0.0356 (10)	0.50
H18A	0.2755	0.0908	0.3692	0.043*	0.50
H18B	0.1433	0.1362	0.3174	0.043*	0.50
C19A	0.28856 (18)	0.17707 (11)	0.27285 (15)	0.0305 (3)	0.50
H19A	0.3703	0.1875	0.3351	0.037*	0.50
H19B	0.2348	0.2222	0.2567	0.037*	0.50
C16B	0.26125 (19)	0.04707 (11)	0.16388 (15)	0.0317 (4)	0.50
H16C	0.1903	0.0312	0.0963	0.038*	0.50
H16D	0.3319	0.0103	0.1821	0.038*	0.50
C17B	0.2106 (4)	0.0498 (2)	0.2547 (3)	0.0359 (8)	0.50
H17C	0.1297	0.0205	0.2327	0.043*	0.50
H17D	0.2753	0.0262	0.3197	0.043*	0.50
C18B	0.1831 (4)	0.12778 (18)	0.2842 (5)	0.0356 (10)	0.50
H18C	0.1874	0.1292	0.3590	0.043*	0.50
H18D	0.0958	0.1443	0.2349	0.043*	0.50
C19B	0.28856 (18)	0.17707 (11)	0.27285 (15)	0.0305 (3)	0.50
H19C	0.3686	0.1729	0.3389	0.037*	0.50
H19D	0.2594	0.2289	0.2660	0.037*	0.50
C20	0.0148 (2)	0.10666 (15)	−0.0424 (3)	0.0766 (11)	
H20A	−0.0670	0.1289	−0.0908	0.115*	
H20B	−0.0007	0.0773	0.0125	0.115*	
H20C	0.0490	0.0749	−0.0839	0.115*	
C21	0.0218 (2)	0.23610 (18)	0.0609 (2)	0.0561 (7)	
H21A	−0.0599	0.2429	−0.0015	0.084*	
H21B	0.0621	0.2841	0.0858	0.084*	
H21C	0.0045	0.2120	0.1190	0.084*	
C22	0.1375 (2)	0.22637 (14)	−0.09285 (17)	0.0415 (5)	
H22A	0.0492	0.2385	−0.1427	0.062*	
H22B	0.1786	0.1947	−0.1293	0.062*	
H22C	0.1881	0.2717	−0.0693	0.062*	

### Atomic displacement parameters (Å^2^)

**Table:**

	*U*^11^	*U*^22^	*U*^33^	*U*^12^	*U*^13^	*U*^23^
Ir1	0.01893 (3)	0.02738 (4)	0.02017 (3)	0.00079 (2)	0.00933 (2)	0.00218 (2)
P1	0.01929 (19)	0.0272 (2)	0.0285 (2)	−0.00024 (16)	0.00746 (16)	0.00237 (17)
N1	0.0266 (7)	0.0315 (8)	0.0238 (7)	−0.0045 (6)	0.0080 (6)	0.0011 (6)
N2	0.0303 (8)	0.0394 (9)	0.0256 (7)	−0.0122 (6)	0.0122 (6)	−0.0033 (6)
N3	0.0234 (7)	0.0362 (8)	0.0225 (7)	−0.0001 (6)	0.0098 (6)	−0.0009 (6)
N4	0.0251 (7)	0.0427 (9)	0.0228 (7)	−0.0014 (6)	0.0137 (6)	−0.0009 (6)
N5	0.0214 (7)	0.0345 (8)	0.0225 (7)	0.0022 (6)	0.0096 (5)	−0.0004 (6)
N6	0.0198 (7)	0.0476 (10)	0.0235 (7)	−0.0001 (6)	0.0096 (6)	−0.0025 (6)
B1	0.0238 (9)	0.0479 (12)	0.0254 (9)	−0.0078 (8)	0.0134 (8)	−0.0018 (8)
C1	0.0451 (13)	0.0331 (11)	0.0691 (18)	0.0079 (9)	0.0169 (13)	0.0008 (10)
C2	0.0351 (10)	0.0329 (9)	0.0276 (9)	−0.0014 (8)	0.0047 (8)	0.0022 (7)
C3	0.0493 (12)	0.0316 (10)	0.0318 (10)	−0.0108 (9)	0.0073 (9)	0.0005 (8)
C4	0.0473 (12)	0.0437 (11)	0.0305 (10)	−0.0227 (10)	0.0148 (9)	−0.0029 (8)
C5	0.0594 (17)	0.0677 (18)	0.0575 (16)	−0.0358 (14)	0.0295 (14)	−0.0074 (13)
C6	0.0388 (11)	0.0524 (13)	0.0364 (11)	−0.0083 (10)	0.0094 (9)	−0.0112 (10)
C7	0.0289 (9)	0.0366 (10)	0.0236 (8)	0.0043 (7)	0.0076 (7)	−0.0016 (7)
C8	0.0378 (10)	0.0473 (11)	0.0249 (8)	0.0051 (9)	0.0144 (8)	−0.0057 (8)
C9	0.0358 (10)	0.0483 (11)	0.0259 (9)	0.0044 (9)	0.0190 (8)	0.0007 (8)
C10	0.0542 (15)	0.0689 (17)	0.0444 (13)	−0.0078 (12)	0.0371 (12)	−0.0049 (11)
C11	0.0430 (11)	0.0474 (12)	0.0287 (9)	0.0013 (9)	0.0080 (8)	0.0111 (9)
C12	0.0293 (9)	0.0364 (9)	0.0229 (8)	0.0066 (7)	0.0074 (7)	−0.0002 (7)
C13	0.0273 (9)	0.0522 (12)	0.0267 (9)	0.0113 (8)	0.0036 (7)	−0.0031 (8)
C14	0.0214 (8)	0.0593 (13)	0.0288 (9)	0.0030 (8)	0.0082 (7)	−0.0085 (9)
C15	0.0223 (10)	0.100 (2)	0.0496 (15)	−0.0031 (11)	0.0125 (10)	−0.0062 (13)
C16A	0.0358 (9)	0.0280 (8)	0.0341 (9)	0.0021 (7)	0.0167 (7)	0.0057 (7)
C17A	0.033 (2)	0.0384 (19)	0.040 (2)	−0.0027 (15)	0.0187 (18)	0.0128 (13)
C18A	0.017 (2)	0.0585 (14)	0.038 (2)	0.0027 (16)	0.018 (2)	0.0024 (14)
C19A	0.0327 (9)	0.0356 (8)	0.0287 (9)	0.0026 (7)	0.0180 (7)	0.0002 (7)
C16B	0.0358 (9)	0.0280 (8)	0.0341 (9)	0.0021 (7)	0.0167 (7)	0.0057 (7)
C17B	0.034 (2)	0.0433 (12)	0.032 (2)	−0.0098 (16)	0.0145 (17)	0.0081 (19)
C18B	0.017 (2)	0.0585 (14)	0.038 (2)	0.0027 (16)	0.018 (2)	0.0024 (14)
C19B	0.0327 (9)	0.0356 (8)	0.0287 (9)	0.0026 (7)	0.0180 (7)	0.0002 (7)
C20	0.0219 (10)	0.0399 (13)	0.136 (3)	−0.0061 (9)	−0.0040 (14)	−0.0021 (16)
C21	0.0346 (11)	0.087 (2)	0.0450 (13)	0.0244 (12)	0.0136 (10)	−0.0045 (13)
C22	0.0356 (10)	0.0567 (13)	0.0292 (9)	0.0088 (9)	0.0093 (8)	0.0135 (9)

### Geometric parameters (Å, º)

**Table:**

Ir1—C19A	2.0780 (18)	C9—C10	1.492 (3)
Ir1—C16A	2.0871 (19)	C10—H10A	0.9800
Ir1—N5	2.1781 (14)	C10—H10B	0.9800
Ir1—N3	2.2233 (15)	C10—H10C	0.9800
Ir1—P1	2.2381 (5)	C11—C12	1.489 (3)
Ir1—N1	2.2590 (15)	C11—H11A	0.9800
P1—C22	1.803 (2)	C11—H11B	0.9800
P1—C20	1.818 (2)	C11—H11C	0.9800
P1—C21	1.830 (2)	C12—C13	1.395 (3)
N1—C2	1.342 (3)	C13—C14	1.369 (3)
N1—N2	1.373 (2)	C13—H13	0.9500
N2—C4	1.353 (3)	C14—C15	1.492 (3)
N2—B1	1.520 (3)	C15—H15A	0.9800
N3—C7	1.340 (2)	C15—H15B	0.9800
N3—N4	1.377 (2)	C15—H15C	0.9800
N4—C9	1.357 (2)	C16A—C17A	1.537 (2)
N4—B1	1.531 (3)	C16A—H16A	0.9900
N5—C12	1.344 (2)	C16A—H16B	0.9900
N5—N6	1.379 (2)	C17A—C18A	1.536 (2)
N6—C14	1.354 (2)	C17A—H17A	0.9900
N6—B1	1.544 (3)	C17A—H17B	0.9900
B1—H1	1.0000	C18A—C19A	1.536 (2)
C1—C2	1.489 (4)	C18A—H18A	0.9900
C1—H1A	0.9800	C18A—H18B	0.9900
C1—H1B	0.9800	C19A—H19A	0.9900
C1—H1C	0.9800	C19A—H19B	0.9900
C2—C3	1.399 (3)	C17B—C18B	1.536 (2)
C3—C4	1.366 (4)	C17B—H17C	0.9900
C3—H3	0.9500	C17B—H17D	0.9900
C4—C5	1.512 (3)	C18B—H18C	0.9900
C5—H5A	0.9800	C18B—H18D	0.9900
C5—H5B	0.9800	C20—H20A	0.9800
C5—H5C	0.9800	C20—H20B	0.9800
C6—C7	1.487 (3)	C20—H20C	0.9800
C6—H6A	0.9800	C21—H21A	0.9800
C6—H6B	0.9800	C21—H21B	0.9800
C6—H6C	0.9800	C21—H21C	0.9800
C7—C8	1.395 (3)	C22—H22A	0.9800
C8—C9	1.367 (3)	C22—H22B	0.9800
C8—H8	0.9500	C22—H22C	0.9800
			
C19A—Ir1—C16A	82.12 (8)	N4—C9—C8	107.64 (17)
C19A—Ir1—N5	91.29 (7)	N4—C9—C10	123.5 (2)
C16A—Ir1—N5	91.71 (7)	C8—C9—C10	128.87 (19)
C19A—Ir1—N3	172.64 (7)	C9—C10—H10A	109.5
C16A—Ir1—N3	96.06 (7)	C9—C10—H10B	109.5
N5—Ir1—N3	81.62 (5)	H10A—C10—H10B	109.5
C19A—Ir1—P1	93.22 (6)	C9—C10—H10C	109.5
C16A—Ir1—P1	89.71 (6)	H10A—C10—H10C	109.5
N5—Ir1—P1	175.42 (4)	H10B—C10—H10C	109.5
N3—Ir1—P1	93.90 (4)	C12—C11—H11A	109.5
C19A—Ir1—N1	92.31 (7)	C12—C11—H11B	109.5
C16A—Ir1—N1	173.83 (6)	H11A—C11—H11B	109.5
N5—Ir1—N1	85.75 (6)	C12—C11—H11C	109.5
N3—Ir1—N1	89.14 (6)	H11A—C11—H11C	109.5
P1—Ir1—N1	93.25 (4)	H11B—C11—H11C	109.5
C22—P1—C20	101.00 (15)	N5—C12—C13	109.95 (18)
C22—P1—C21	103.01 (12)	N5—C12—C11	126.10 (17)
C20—P1—C21	96.38 (14)	C13—C12—C11	123.96 (18)
C22—P1—Ir1	111.42 (7)	C14—C13—C12	106.23 (17)
C20—P1—Ir1	119.95 (9)	C14—C13—H13	126.9
C21—P1—Ir1	121.84 (8)	C12—C13—H13	126.9
C2—N1—N2	105.75 (16)	N6—C14—C13	107.86 (18)
C2—N1—Ir1	137.67 (14)	N6—C14—C15	123.8 (2)
N2—N1—Ir1	116.49 (12)	C13—C14—C15	128.3 (2)
C4—N2—N1	110.26 (18)	C14—C15—H15A	109.5
C4—N2—B1	129.95 (18)	C14—C15—H15B	109.5
N1—N2—B1	119.62 (15)	H15A—C15—H15B	109.5
C7—N3—N4	106.05 (15)	C14—C15—H15C	109.5
C7—N3—Ir1	138.98 (13)	H15A—C15—H15C	109.5
N4—N3—Ir1	114.84 (11)	H15B—C15—H15C	109.5
C9—N4—N3	109.95 (16)	C17A—C16A—Ir1	111.13 (19)
C9—N4—B1	127.88 (17)	C17A—C16A—H16A	109.4
N3—N4—B1	121.00 (14)	Ir1—C16A—H16A	109.4
C12—N5—N6	106.05 (15)	C17A—C16A—H16B	109.4
C12—N5—Ir1	138.20 (13)	Ir1—C16A—H16B	109.4
N6—N5—Ir1	115.71 (11)	H16A—C16A—H16B	108.0
C14—N6—N5	109.89 (16)	C18A—C17A—C16A	105.5 (3)
C14—N6—B1	128.16 (17)	C18A—C17A—H17A	110.6
N5—N6—B1	121.92 (15)	C16A—C17A—H17A	110.6
N2—B1—N4	111.05 (16)	C18A—C17A—H17B	110.6
N2—B1—N6	109.40 (15)	C16A—C17A—H17B	110.6
N4—B1—N6	109.86 (17)	H17A—C17A—H17B	108.8
N2—B1—H1	108.8	C17A—C18A—C19A	114.6 (3)
N4—B1—H1	108.8	C17A—C18A—H18A	108.6
N6—B1—H1	108.8	C19A—C18A—H18A	108.6
C2—C1—H1A	109.5	C17A—C18A—H18B	108.6
C2—C1—H1B	109.5	C19A—C18A—H18B	108.6
H1A—C1—H1B	109.5	H18A—C18A—H18B	107.6
C2—C1—H1C	109.5	C18A—C19A—Ir1	111.3 (2)
H1A—C1—H1C	109.5	C18A—C19A—H19A	109.4
H1B—C1—H1C	109.5	Ir1—C19A—H19A	109.4
N1—C2—C3	110.2 (2)	C18A—C19A—H19B	109.4
N1—C2—C1	124.98 (19)	Ir1—C19A—H19B	109.4
C3—C2—C1	124.8 (2)	H19A—C19A—H19B	108.0
C4—C3—C2	105.85 (19)	C18B—C17B—H17C	108.7
C4—C3—H3	127.1	C18B—C17B—H17D	108.7
C2—C3—H3	127.1	H17C—C17B—H17D	107.6
N2—C4—C3	107.92 (19)	C17B—C18B—H18C	110.6
N2—C4—C5	122.5 (2)	C17B—C18B—H18D	110.6
C3—C4—C5	129.5 (2)	H18C—C18B—H18D	108.7
C4—C5—H5A	109.5	P1—C20—H20A	109.5
C4—C5—H5B	109.5	P1—C20—H20B	109.5
H5A—C5—H5B	109.5	H20A—C20—H20B	109.5
C4—C5—H5C	109.5	P1—C20—H20C	109.5
H5A—C5—H5C	109.5	H20A—C20—H20C	109.5
H5B—C5—H5C	109.5	H20B—C20—H20C	109.5
C7—C6—H6A	109.5	P1—C21—H21A	109.5
C7—C6—H6B	109.5	P1—C21—H21B	109.5
H6A—C6—H6B	109.5	H21A—C21—H21B	109.5
C7—C6—H6C	109.5	P1—C21—H21C	109.5
H6A—C6—H6C	109.5	H21A—C21—H21C	109.5
H6B—C6—H6C	109.5	H21B—C21—H21C	109.5
N3—C7—C8	110.04 (17)	P1—C22—H22A	109.5
N3—C7—C6	125.19 (18)	P1—C22—H22B	109.5
C8—C7—C6	124.74 (18)	H22A—C22—H22B	109.5
C9—C8—C7	106.32 (17)	P1—C22—H22C	109.5
C9—C8—H8	126.8	H22A—C22—H22C	109.5
C7—C8—H8	126.8	H22B—C22—H22C	109.5
			
C19A—Ir1—P1—C22	139.06 (10)	C9—N4—B1—N6	−117.7 (2)
C16A—Ir1—P1—C22	−138.85 (10)	N3—N4—B1—N6	48.6 (2)
N3—Ir1—P1—C22	−42.79 (10)	C14—N6—B1—N2	−116.6 (2)
N1—Ir1—P1—C22	46.56 (10)	N5—N6—B1—N2	65.6 (2)
C19A—Ir1—P1—C20	−103.39 (16)	C14—N6—B1—N4	121.2 (2)
C16A—Ir1—P1—C20	−21.30 (16)	N5—N6—B1—N4	−56.6 (2)
N3—Ir1—P1—C20	74.76 (16)	N2—N1—C2—C3	−0.1 (2)
N1—Ir1—P1—C20	164.11 (16)	Ir1—N1—C2—C3	176.12 (14)
C19A—Ir1—P1—C21	17.18 (14)	N2—N1—C2—C1	179.2 (2)
C16A—Ir1—P1—C21	99.27 (14)	Ir1—N1—C2—C1	−4.5 (3)
N3—Ir1—P1—C21	−164.67 (13)	N1—C2—C3—C4	0.3 (2)
N1—Ir1—P1—C21	−75.32 (13)	C1—C2—C3—C4	−179.0 (2)
C19A—Ir1—N1—C2	−40.51 (19)	N1—N2—C4—C3	0.4 (2)
N5—Ir1—N1—C2	−131.63 (19)	B1—N2—C4—C3	−174.72 (18)
N3—Ir1—N1—C2	146.71 (19)	N1—N2—C4—C5	178.7 (2)
P1—Ir1—N1—C2	52.85 (19)	B1—N2—C4—C5	3.6 (3)
C19A—Ir1—N1—N2	135.43 (13)	C2—C3—C4—N2	−0.4 (2)
N5—Ir1—N1—N2	44.31 (12)	C2—C3—C4—C5	−178.6 (2)
N3—Ir1—N1—N2	−37.35 (12)	N4—N3—C7—C8	−1.2 (2)
P1—Ir1—N1—N2	−131.21 (11)	Ir1—N3—C7—C8	174.17 (15)
C2—N1—N2—C4	−0.2 (2)	N4—N3—C7—C6	176.69 (19)
Ir1—N1—N2—C4	−177.32 (13)	Ir1—N3—C7—C6	−7.9 (3)
C2—N1—N2—B1	175.50 (16)	N3—C7—C8—C9	1.1 (2)
Ir1—N1—N2—B1	−1.7 (2)	C6—C7—C8—C9	−176.8 (2)
C16A—Ir1—N3—C7	37.8 (2)	N3—N4—C9—C8	−0.2 (2)
N5—Ir1—N3—C7	128.7 (2)	B1—N4—C9—C8	167.3 (2)
P1—Ir1—N3—C7	−52.3 (2)	N3—N4—C9—C10	179.1 (2)
N1—Ir1—N3—C7	−145.5 (2)	B1—N4—C9—C10	−13.4 (3)
C16A—Ir1—N3—N4	−147.08 (13)	C7—C8—C9—N4	−0.5 (2)
N5—Ir1—N3—N4	−56.23 (12)	C7—C8—C9—C10	−179.7 (2)
P1—Ir1—N3—N4	122.79 (12)	N6—N5—C12—C13	0.3 (2)
N1—Ir1—N3—N4	29.60 (12)	Ir1—N5—C12—C13	−177.23 (15)
C7—N3—N4—C9	0.9 (2)	N6—N5—C12—C11	−179.78 (19)
Ir1—N3—N4—C9	−175.78 (13)	Ir1—N5—C12—C11	2.7 (3)
C7—N3—N4—B1	−167.66 (17)	N5—C12—C13—C14	0.5 (2)
Ir1—N3—N4—B1	15.7 (2)	C11—C12—C13—C14	−179.4 (2)
C19A—Ir1—N5—C12	45.3 (2)	N5—N6—C14—C13	1.3 (2)
C16A—Ir1—N5—C12	−36.8 (2)	B1—N6—C14—C13	−176.71 (19)
N3—Ir1—N5—C12	−132.7 (2)	N5—N6—C14—C15	−176.8 (2)
N1—Ir1—N5—C12	137.5 (2)	B1—N6—C14—C15	5.2 (3)
C19A—Ir1—N5—N6	−132.07 (13)	C12—C13—C14—N6	−1.1 (2)
C16A—Ir1—N5—N6	145.77 (13)	C12—C13—C14—C15	176.9 (2)
N3—Ir1—N5—N6	49.90 (12)	C19A—Ir1—C16A—C17A	29.0 (2)
N1—Ir1—N5—N6	−39.86 (12)	N5—Ir1—C16A—C17A	120.0 (2)
C12—N5—N6—C14	−1.0 (2)	N3—Ir1—C16A—C17A	−158.2 (2)
Ir1—N5—N6—C14	177.18 (13)	P1—Ir1—C16A—C17A	−64.3 (2)
C12—N5—N6—B1	177.15 (17)	Ir1—C16A—C17A—C18A	−42.2 (4)
Ir1—N5—N6—B1	−4.7 (2)	C16A—C17A—C18A—C19A	36.2 (5)
C4—N2—B1—N4	−123.3 (2)	C17A—C18A—C19A—Ir1	−14.3 (4)
N1—N2—B1—N4	62.0 (2)	C16A—Ir1—C19A—C18A	−8.1 (2)
C4—N2—B1—N6	115.2 (2)	N5—Ir1—C19A—C18A	−99.6 (2)
N1—N2—B1—N6	−59.4 (2)	P1—Ir1—C19A—C18A	81.2 (2)
C9—N4—B1—N2	121.1 (2)	N1—Ir1—C19A—C18A	174.6 (2)
N3—N4—B1—N2	−72.6 (2)		
